# Marfan’s syndrome with anterior sacral pseudomeningocele

**DOI:** 10.1093/omcr/omac139

**Published:** 2022-12-16

**Authors:** Christelle Nguyen, Guillaume Jondeau

**Affiliations:** Université Paris Cité, Faculté de Santé, UFR de Médecine, Paris, France; AP-HP.Centre-Université Paris Cité, Hôpital Cochin, Service de Rééducation et de Réadaptation de l’Appareil Locomoteur et des Pathologies du Rachis, Paris, France; INSERM UMRS-1124, Toxicité Environnementale, Cibles Thérapeutiques, Signalisation Cellulaire et Biomarqueurs (T3S), Campus Saint-Germain-des-Prés, Paris, France; Université Paris Cité, Faculté de Santé, UFR de Médecine, Paris, France; AP-HP.Nord-Université Paris Cité, Hôpital Bichat, Centre de Référence pour le syndrome de Marfan et apparentés, VASCERN HCP, Paris, France; INSERM U1148, Laboratory for Vascular Translational Science, Hôpital Bichat, Paris, France

**Figure 1:**
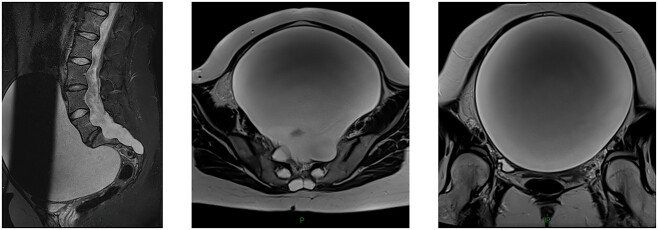
Lumbar magnetic resonance imaging in T2-weighted sequence showing an anterior sacral pseudomeningocele in a 27-year-old woman with Marfan syndrom. Left panel: sagittal view; Middle panel: axial view and Right panel: coronal view.

A 27-year-old woman, who had a history of Marfan syndrome with mutation in the *FBN1* gene, presented with mild chronic low back pain occurring in upright position. Symptoms had spontaneously improved in the previous year. She had no bladder, bowel or neurological complaints. Physical examination revealed Marfan’s skeletal features including arachnodactyly, platypodia, scoliosis, joint hypermobility and arched palate. Lumbar spine Magnetic resonance imaging showed an anterior sacral pseudomeningocele emanated from a bone defect along the anterior margin of the sacrum, extending to the L3–L4 interspace and the anterior abdominal wall **(**Fig. 1). After multidisciplinary team meeting, conservative management was offered. At 3-year follow-up, symptoms were stable.

Dural ectasia is a frequent feature of Marfan syndrome, but progression to anterior sacral pseudomeningocele is uncommon [1]. Conservative management is indicated for paucisymptomatic stable lesions [2]. Neurosurgical approach may be considered for symptomatic lesions, particularly in cases of bowel and bladder involvements or neurological deficits [2, 3].
